# Myths and methodologies: Assessment of dynamic cerebral autoregulation by the mean flow index

**DOI:** 10.1113/EP091327

**Published:** 2024-02-20

**Authors:** Markus Harboe Olsen, Christian Gunge Riberholt, Ronan M. G. Berg, Kirsten Møller

**Affiliations:** ^1^ Department of Neuroanaesthesiology, The Neuroscience Centre Copenhagen University Hospital − Rigshospitalet Copenhagen Denmark; ^2^ Department of Brain and Spinal Cord Injury, The Neuroscience Centre Copenhagen University Hospital − Rigshospitalet Copenhagen Denmark; ^3^ Department of Clinical Physiology and Nuclear Medicine Copenhagen University Hospital − Rigshospitalet Copenhagen Denmark; ^4^ Centre for Physical Activity Research Copenhagen University Hospital − Rigshospitalet Copenhagen Denmark; ^5^ Department of Biomedical Sciences, Faculty of Health and Medical Sciences University of Copenhagen Copenhagen Denmark; ^6^ Neurovascular Research Laboratory, Faculty of Life Sciences and Education University of South Wales Pontypridd UK; ^7^ Department of Clinical Medicine, Faculty of Health and Medical Sciences University of Copenhagen Copenhagen Denmark

**Keywords:** cerebral haemodynamics, physiolometrics, reliability, transcranial doppler, validity

## Abstract

The mean flow index—usually referred to as Mx—has been used for assessing dynamic cerebral autoregulation (dCA) for almost 30 years. However, concerns have arisen regarding methodological consistency, construct and criterion validity, and test–retest reliability. Methodological nuances, such as choice of input (cerebral perfusion pressure, invasive or non‐invasive arterial pressure), pre‐processing approach and artefact handling, significantly influence mean flow index values, and previous studies correlating mean flow index with other established dCA metrics are confounded by inherent methodological flaws like heteroscedasticity, while the mean flow index also fails to discriminate individuals with presumed intact versus impaired dCA (discriminatory validity), and its prognostic performance (predictive validity) across various conditions remains inconsistent. The test–retest reliability, both within and between days, is generally poor. At present, no single approach for data collection or pre‐processing has proven superior for obtaining the mean flow index, and caution is advised in the further use of mean flow index‐based measures for assessing dCA, as current evidence does not support their clinical application.

## Abstract (highlights)

- **What is the topic of this review?**
  The validity and reliability of mean flow index‐based measures for assessing dynamic cerebral autoregulation (dCA) in humans.
- **What advances does it highlight?**
  Current evidence does not support the clinical application of mean flow index‐based measures, warranting caution in their further use for dCA assessment.

## 1 INTRODUCTION

The mean flow index—usually referred to as Mx—is a commonly used correlation‐based index of dynamic cerebral autoregulation (dCA) that was introduced in 1996 (Czosnyka et al., 1996). In contrast to static cerebral autoregulation—which assumes that the variable for pressure (e.g., arterial blood pressure; ABP) and cerebral blood flow (CBF) are in a steady state (Panerai et al., 1998)—dCA refers to the immediate cerebrovascular responses that occur with rapid changes in ABP (Aaslid et al., 1989; Hea Van Beek et al., 2008; Panerai et al., 1998). In principle, the mean flow index provides information about dCA in the time domain, that is, how quickly the cerebrovasculature responds to buffer the impact of acute fluctuations in the input, being either cerebral perfusion pressure (CPP), invasive ABP or non‐invasive ABP (Lavinio et al., 2007; Liu et al., 2015; Petersen et al., 2014), on the output, CBF, usually assessed by transcranial Doppler ultrasound (TCD)‐based middle cerebral artery blood flow velocity (MCAv) (Czosnyka et al., 1996; Reinhard et al., 2003).

There is a large interest in developing methods and indices of dCA that are feasible and applicable in the clinical setting, because it is thought to be impaired in a wide array of both acute and chronic conditions, such as stroke and obstructive sleep apnoea (Nasr et al., 2009; Reinhard, Gerds et al., 2008), and in patients with acute brain injury (Svedung Wettervik et al., 2021). Thus, clinical assessments of dCA have the potential to diagnose patients with complex symptomatology, forewarn clinical worsening and potentially personalize neuroprotective treatments (Claassen et al., 2021; Czosnyka et al., 2009). In this context, the mean flow index and derived indices are attractive, because they are relatively easy to obtain and interpret, and furthermore permit the continuous monitoring of dCA. Thus, mean flow index is considered a potentially valuable clinical tool for prognostic stratification, particularly in the neurointensive care setting, and commercially available software has been developed for its integration in multimodal neuromonitoring (Klein et al., 2019; Vitt et al., 2023).

As mean flow index‐based methods are becoming widely implemented clinically, it is necessary to critically evaluate the ‘physiolometrics’, that is, the validity and reliability, of the methodology (Hartmann et al., 2023). In the present paper, we will uncover the different methodological approaches for calculating mean flow index‐based measures. We will, furthermore, systematically evaluate (1) their construct validity, that is, to what extent mean flow index‐based measures behave as expected if they truly reflect dCA, including the ability to distinguish individuals that presumably have intact versus those with impaired dCA (discriminatory validity); (2) their criterion validity, that is, how well they agree with other established measures of dCA (concurrent validity) and also how they may inform prognosis (predictive validity); and (3) their test–retest reliability, that is, the consistency of mean flow index‐based measures in a given population, considering both repeatability (reliability under identical experimental conditions) and reproducibility (reliability between different experimental conditions).

## 2 MEASUREMENT PRINCIPLE AND TERMINOLOGY

A mean flow index is based on waveform recordings of the input (CPP, invasive ABP or non‐invasive ABP) and, typically, TCD‐based MCAv. In the present paper, the term Mxc is used to designate mean flow index based on CPP, whereas Mxa is used for mean flow index based on invasive ABP and nMxa is used when based on non‐invasive ABP, whereas the term ‘mean flow index’ will be used to refer to these three indices more generally.

**FIGURE 1:**
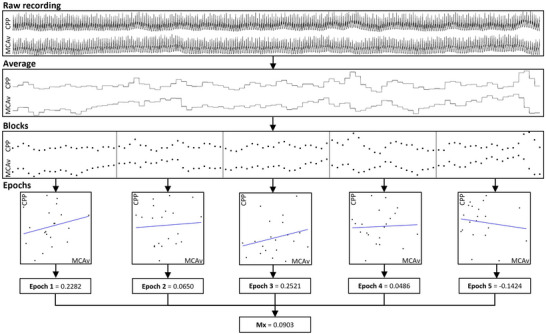
The process of calculating the mean flow index from a raw recording (Olsen, Riberholt, Mehlsen et al., 2022). CPP, cerebral perfusion pressure; MCAv, middle cerebral artery blood flow velocity.

**FIGURE 2:**
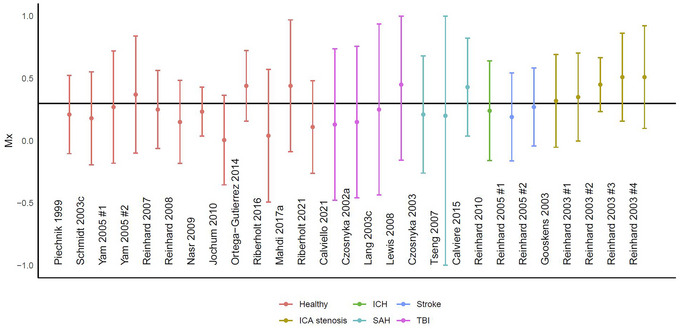
An overview of previous studies stratified by study and diagnosis (colour) (Calviello et al., 2021; Calviere et al., 2015; Czosnyka et al., 2002, 2003; Gooskens et al., 2003; Jochum et al., 2010; Lang et al., 2003c; Lewis et al., 2008; Mahdi, Nikolic, Birch, Olufsen et al., 2017; Nasr et al., 2009; Ortega‐Gutierrez et al., 2014; Piechnik et al., 1999; Reinhard et al., 2003, 2005, 2007, Reinhard, Waldkircher et al., 2008, 2010; Riberholt et al., 2016, 2021; Schmidt et al., 2003; Tseng et al., 2007; Yam et al., 2005). The data are presented as mean (95% CI); the black horizontal line depicts the conventional threshold between intact and impaired cerebral autoregulation of 0.3. For the studies with multiple presented values the first, the left, the baseline, or the ipsilateral is chosen. ICH: Intracerebral haemorrhage; ICA: internal carotid artery; SAH: subarachnoid haemorrhage; TBI: traumatic brain injury.

In general, the following procedure is followed for deriving a mean flow index regardless of underlying blood pressure source. The waveform recordings are averaged over a period of 3 to 10 s (called ‘blocks’). These blocks are split into groups of 20 to 40 (called ‘epochs’). The blocks of pressure and MCAv measurements are then correlated using Pearson's correlation coefficient for every epoch. Recordings with more than one epoch, and thereby more than one correlation coefficient, are then averaged into the final result (Czosnyka et al., 1996) (Figure 1). The resultant mean flow index ranges from −1 to +1, where a value close to +1 will indicate that fluctuations in CBF follow the input closely, and thus that dCA is impaired, and vice versa for low positive and negative values (Czosnyka et al., 1996, 2002; Lang et al., 2002). In some cases, a threshold is set above which the mean flow index is considered abnormal, so that dCA can be classified dichotomously as intact or impaired. This threshold has conventionally been set at either 0.30 or 0.45 (Olsen et al., 2021) (Figure 2; Ortega‐Gutierrez et al., 2014; Reinhard et al., 2007; Yam et al., 2005).

Mean flow index‐based measures can be calculated in several different ways, depending on choices made in terms of pre‐processing of data, including handling of artefacts, as well as block and epoch sizes. In the present paper, the pre‐processing settings for a given mean flow index measure are reported as a three‐figure code, when relevant: block length (seconds)–epoch length (seconds)–segment overlap length (seconds; F = no overlap). For example, Mxc (3–60–F) means that the input is CPP, the block length is 3 s, and the epoch length is 60 s with no segment overlap (Olsen, Riberholt, Mehlsen et al., 2022).

## 3 METHODOLOGICAL APPROACHES

According to a recent systematic review that included 128 studies (Olsen, Riberholt, Mehlsen et al., 2022), Mxc was reported in 31 (24%), Mxa in 37 (29%), nMxa in 40 (31%) studies, while multiple indices were used in 11 (9%) studies, and the exact approach was unclear in 9 (7%) studies. In general, Mxa, Mxc, and nMxa provide different results. Hence, Mxa generally results in higher values than Mxc and nMxa (Lavinio et al., 2007; Olsen, Capion et al., 2022), and although the two latter generally provide values in the same range (Liu et al., 2015; Olsen, Capion et al., 2022; Schmidt et al., 2003), they do so with large intra‐individual variation, particularly when intracranial pressure increases (Liu et al., 2016).

Among the 128 studies in the systematic review, 6–240–F, 10–300–F and 10–300–60 were the most commonly used approaches for data processing; however, the specific details on pre‐processing were reported in less than half of the included papers (Olsen, Riberholt, Mehlsen et al., 2022). The blocks were predominantly non‐overlapping with a duration from 3 to 10 s. Similarly, the epoch sizes ranged from including 10 to 60 blocks. The epochs overlapped in 21 studies, typically by 1 to 6 blocks between each new calculation. These choices made for data pre‐processing are crucial for the final mean flow index value, as it is obtained by different approaches that agree poorly even when based on the same input (Olsen et al., 2021), which may to some extent reflect that invasive and non‐invasive ABP measurements in themselves show poor agreement (Kamboj et al., 2021; Kim et al., 2014; Olsen, Riberholt, Capion et al., 2022). In the same systematic review, the removal of artefacts was also only described in less than half of the included studies (Olsen et al., 2022). In fact, only one study defined an upper limit of 10% for the acceptable prevalence of artefacts before exclusion of data (Crippa et al., 2018). In healthy volunteers, the amount of artefacts has been shown to markedly influence the final results for Mxa, and to reduce the agreement between the results achieved by various approaches (3–60–F, 6–240–F, 10–300–F and 10–300–60) (Olsen et al., 2021). In fact, one modelling study showed that increasing the amount of noise systematically increased nMxa (10–300–F) (Liu et al., 2020). Finally, the optimal recording duration for achieving a stable nMxa value (not accounting for pre‐processing methodology) has previously been proposed to be 6 min (Mahdi, Nikolic, Birch, & Payne, 2017), but at least 7 (5%) of the 128 studies included in the systematic review used shorter recording times (Olsen, Riberholt, Mehlsen et al., 2022).

In summary, the existing literature on the mean flow index presents considerable challenges for interpretation due to the use of varied input types (CPP, invasive ABP, or non‐invasive ABP), as well as divergent methodologies for data pre‐processing and artefact handling. These inconsistencies introduce substantial variability into the resulting mean flow index‐based measures, thereby complicating the comparison and synthesis of published studies.

## 4 CONSTRUCT VALIDITY

If mean flow index‐based measures were valid measures of dCA, they should respond to stimuli that are known to affect the cerebral pressure–flow relationship when assessed by other methods. Hypo‐ and hypercapnia are known to have a profound effect on CBF, and have been reported to enhance and impair dCA, respectively, according to the thigh cuff deflation technique and transfer function analysis (Aaslid et al., 1989; Edwards et al., 2002; Maggio et al., 2013, 2014; Zhang et al., 1998). In patients with traumatic brain injury (TBI), after a 20‐min recording with stable end‐tidal $P_{{\mathrm{CO}}_{2}}$, moderate hypocapnia was obtained by increasing the minute ventilation by 15–20% on the mechanical ventilator, which led to a decrease in $P_{{\mathrm{aCO}}_{2}}$ of 0.8 kPa and in Mxc (10–240–F) of ∼0.15 (Haubrich et al., 2011). Furthermore, in patients with internal carotid artery occlusive disease, breathing hypercapnic air (5% CO_2_) resulted in an increase in end‐tidal $P_{{\mathrm{CO}}_{2}}$ of ∼1.3 kPa and a corresponding increase in nMxa (5–180–F) of ∼0.13 (Gooskens et al., 2003). Finally, decreasing end‐tidal $P_{{\mathrm{CO}}_{2}}$ by ∼0.6 kPa by voluntary hyperventilation in healthy volunteers caused a ∼0.13 decrease in nMxa (10–300–F) (Uryga et al., 2017). Finally, the side of insonation seems to influence Mxc (Haubrich et al., 2011, 2012; Schmidt et al., 2003; Tseng et al., 2007), Mxa (Joshi et al., 2010; Lang et al., 2003c; Lewis et al., 2008; Soehle et al., 2004) and nMxa (Gooskens et al., 2003; Hori et al., 2015; Jochum et al., 2010; Ortega‐Gutierrez et al., 2014; Petersen et al., 2014; Reinhard et al., 2003, 2004, 2005, 2007, 2010, Reinhard, Rutsch, Lambeck et al., 2012; Yam et al., 2005). Thus, in general higher values were measured ipsilateral to the injury; however, side differences were also reported in healthy volunteers (Yam et al., 2005) and patients without unilateral damage (Jochum et al., 2010; Reinhard et al., 2007). Of note, our expected behaviour is based on other methods, which are in their own way flawed or not thoroughly investigated in terms of physiolometrics (Claassen et al., 2021; Olsen, Riberholt, Plovsing et al., 2022), and furthermore do not correlate well with each other (Caldas et al., 2022; Ortega‐Gutierrez et al., 2014; Pochard et al., 2020; Tzeng et al., 2012). For instance, the autoregulatory index (ARI) has poor test–retest reliability (Lee et al., 2020), the pressure reactivity index (PRx) has only moderate accuracy for predicting all‐cause mortality in patients with severe TBI (Riemann et al., 2020), and transfer function analysis metrics show a questionable ability to discriminate between healthy volunteers and patients with TBI (Olsen, Riberholt, Plovsing et al., 2022).

Another aspect of construct validity is the ability of mean flow index‐based measures to discriminate individuals that are assumed to have normal versus abnormal dCA. In healthy volunteers, impaired cerebral autoregulation based on the suggested dichotomization of the mean flow index at 0.30 was observed in 56% for nMxa (10–300–F) and 74% for nMxa (3–60–F). Even when the dichotomous threshold was set at 0.45, for nMxa 18% (10–300–F) and 40% (3–60–F) of the volunteers were still classified as having impaired dCA (Olsen et al., 2021). Surprisingly, in patients with severe TBI and mixed populations of acute brain injury, the mean values were lower than both the 0.30 and 0.45 threshold (Riberholt et al., 2016; Soehle et al., 2004; Uryga et al., 2018), which should indicate preserved dCA, even when only including those with an unfavourable outcome (Czosnyka et al., 2001; Uryga et al., 2018). This discrepancy is further underlined when trying to ascertain the ability of Mxa (10–300–F) to discriminate between healthy volunteers and patients from the acute phase after TBI, patients in rehabilitation after TBI, and critically ill patients with sepsis (Olsen, Riberholt, Plovsing et al., 2022). In these populations, Mxa performed no better than chance in its ability to discriminate between healthy volunteers and these patient categories, regardless of the approach (3–60–F, 6–240–F, 10–300–F or 10–300–60) (Olsen, Riberholt, Plovsing et al., 2022). Thus, if these mean flow index‐based measures are interpreted as truly reflecting dCA and the set thresholds are appropriate, this would lead to the wrong conclusion that healthy volunteers may frequently exhibit weaker dCA than any of these patient groups (Figure 2).

In conclusion, while mean flow index‐based measures as measures of dCA may exhibit meaningful responses to hypo‐ and hypercapnia, the effect of ageing is inconsistent with other dCA indices, as is their ability to discriminate between individuals with presumed normal and abnormal dCA with very limited consistency across different pre‐processing approaches. Notably, none of the pre‐processing approaches have definitively outperformed the others in terms of construct validity.

## 5 CRITERION VALIDITY

In terms of criterion validity, a key question is to what extent the mean flow index agrees with other established indices of dCA that are obtained concurrently in the same individuals. The mean flow index‐based measures have been reported to correlate with the rate of regulation (RoR) to thigh‐cuff deflation (Lang et al., 2002; Piechnik et al., 1999), PRx (Lang et al., 2003b; Pochard et al., 2020; Schmidt et al., 2012; Zeiler et al., 2017, Zeiler, Cardim et al., 2018, Zeiler, Donnelly et al., 2018), and ARI (Czosnyka et al., 2008; Liu et al., 2020). However, almost all these correlations are likely flawed due to suspected heteroscedasticity (Gollion et al., 2019; Quispe Cornejo et al., 2020), with one of the comparators being categorical (Budohoski, Czosnyka et al., 2012; Czosnyka et al., 1997, 1996; Lang et al., 2003a; Reinhard, Rutsch, & Hetzel et al., 2012; Schmidt et al., 2016, 2016; Tang et al., 2008), or because of mathematical coupling (Aggarwal & Ranganathan, 2016; Schober & Schwarte, 2018). Indices such as RoR (Lang et al., 2002; Piechnik et al., 1999), PRx (Lang et al., 2003b; Pochard et al., 2020; Schmidt et al., 2012; Zeiler et al., 2017, Zeiler, Cardim et al., 2018, Zeiler, Donnelly et al., 2018) and ARI (Czosnyka et al., 2008; Liu et al., 2020) have all been derived using the same underlying data pool; although the correlation identified is mathematically correct, it nevertheless does not further corroborate any underlying physiological associations (Aggarwal & Ranganathan, 2016; Schober & Schwarte, 2018).

Another aspect of criterion validity that will be addressed here is the prognostic performance of mean flow index‐based measures, that is, their predictive validity. In one study, Mxa failed to predict neurological or all‐cause mortality, regardless of pre‐processing approach (6–240–F, 10–300–F and 10–300–60) in sepsis, acute TBI or patients undergoing rehabilitation after acute brain injury (Olsen, Riberholt, Plovsing et al., 2022). A similarly poor predictive value has also been shown by others for both Mxa (10–300–F) and Mx (10–300–F) in patients with TBI (Zeiler et al., 2017); and for Mxa (10–300–10) in patients with subarachnoid haemorrhage (Uryga et al., 2018). However, some studies have shown that Mxa (10–300–10) may predict neurological outcome, albeit with low accuracy, in patients with severe TBI (Budohoski, Reinhard et al., 2012), and with moderate accuracy in patients with acute brain injury (10–300–60) (Schmidt et al., 2016).

In summary, the criterion validity of mean flow index‐based measures raises significant concerns. The previously reported correlations with other established dCA are non‐informative, and their prognostic performance is inconsistent and limited across different conditions and studies.

## 6 TEST–RETEST RELIABILITY

Even if a measure were valid, it would only be meaningful as a biomarker for the prediction of a given clinical outcome if repeated measurements obtained under steady state conditions were similar to such an extent that they did not lead to an entirely different prediction. This may be evaluated by assessing the test–retest reliability, encompassing both repeatability, that is, measurements obtained under identical conditions, and reproducibility, that is, measurements obtained under similar conditions, which may both be affected by either non‐stationarity of the underlying biological signal or by measurement error.

In terms of test–retest reliability specifically focused on same‐session repeatability, the mean flow index obtained with different inputs and using different pre‐processing approaches with non‐overlapping recordings has been reported as exhibiting poor to moderate repeatability in healthy individuals, according to the intraclass correlation coefficient (ICC) (Lorenz et al., 2007; Mahdi, Nikolic, Birch, Olufsen et al., 2017; Olsen et al., 2021). In one of these studies on 46 semi‐supine healthy volunteers comparing nMxa (3–60–F) based on consecutive 5‐min recordings, an ICC of 0.39 (95% CI: 0.08, 0.67) was reported (Lorenz et al., 2007). Similarly, nMxa (not accounting for pre‐processing methodology) was obtained in 20 healthy volunteers during 60 s of sitting and 60 s of free‐standing, and in this study sitting nMxa was reported to be poor (ICC ∼0), while moderate repeatability was reported (ICC ∼0.8) for the standing position (Mahdi, Nikolic, Birch, Olufsen et al., 2017). However, the duration of recordings may also have played a part here as they were shorter than the recording length deemed as the point of stabilization, which was determined to be 6 min by the same authors (Mahdi, Nikolic, Birch, & Payne, 2017). In another study on 48 semi‐supine healthy volunteers, the ICC for Mxa and nMxa was obtained for four of the most widely used pre‐processing approaches (3–60–F, 6–240–F, 10–300–F, 10–300–60), and in all cases, the repeatability was poor (ICC between 0.14 and 0.52) (Olsen et al., 2021).

Other studies have examined the reproducibility of mean flow index‐based measures, here defined as the between‐day test–retest reliability. In a study on 19 healthy volunteers, Ortega‐Gutierrez et al. obtained 10‐min recordings 17 (IQR 5–27) days apart and found poor reproducibility for nMxa (3–60–F), when the side of insonation was both the right (ICC: 0.42, 95%CI: −0.34; 0.73) and the left (ICC: 0.46, 95%CI: 0.02; 0.75) (Ortega‐Gutierrez et al., 2014). Similarly, nMxa obtained ∼23 days apart in 14 healthy volunteers placed in the supine position and during head‐up tilt also provided poor reproducibility (ICC between 0.15 and 0.57), regardless of the pre‐processing approach (3–60–F, 5–150–F or 10–300–F) (Riberholt et al., 2021).

However, although ICC is a widely used metric that can conveniently be used for categorizing reliability as poor, moderate, good, or excellent, it offers an incomplete view of test–retest reliability (Hartmann et al., 2023). Firstly, it is purely a measure of relative reliability, providing insights only into the proportion of measurement error relative to the overall variability of the metric. To obtain a more complete understanding of reliability in the same units as the measure itself, absolute reliability metrics such as bias with limits of agreement and the smallest real difference are essential. Secondly, because ICC is influenced by variations both within and between groups, high inter‐subject variability can inflate the ICC. To adequately assess relative reliability, it is beneficial to complement ICC with other metrics like the coefficient of variation. Measures of both absolute and relative test–retest reliability for Mxa, based on the four most common pre‐processing approaches, are presented in Table 1 (Olsen et al., 2021). These indicate that, irrespective of the pre‐processing method employed, Mxa displays a notably large margin of error in both absolute and relative terms. From these, it is clear that repeated measures of Mxa will lead to entirely different conclusions regarding dCA, thus indicating that it has a limited value as a biomarker (Hartmann et al., 2023; Olsen, Riberholt, Plovsing et al., 2022).

**TABLE 1:** Test–retest reliability of Mxa in healthy volunteers (*n* = 46).

Method	Absolute reliability		Relative reliability	
	Bias (units)	SRD (units)	CV (%)	ICC (%)
3–60–F	0 (−0.6 to 0.6)	0.64 (0.54–0.78)	73.5 (57.9–91.6)	0.25 (−0.01 to 0.48)
6–240–F	0 (−0.5 to 0.5)	0.55 (0.43–0.73)	44.95 (30.1–61.6)	0.14 (−0.34 to 0.56)
10–300–F	0 (−0.4 to 0.4)	0.39 (0.31–0.49)	31.39 (25.7–37.3)	0.52 (0.31 to 0.68)
10–300–60	0 (−0.5 to 0.5)	0.51 (0.43–0.6)	52.84 (42.6–64)	0.4 (0.16 to 0.59)

*Note*: Recalculation from previous study (Olsen et al., 2021). Bias represents the overall bias from Bland–Altmann analysis together with 95% limits of agreement. SRD, smallest real difference—an estimate of the maximal difference there will be between two measurements in 95% of occasions; CV, coefficient of variation—reflects the relationship between the standard deviation within the group and the mean; ICC, intraclass correlation coefficient—depicts the agreement between two measurements (Hartmann et al., 2023).

## 7 PERSPECTIVES

According to the available data on the methodology as well as construct and criterion validity reviewed above, every step from raw data collection to the pre‐processing approach and artefact handling as well as the choice of dichotomous threshold (if any) influences the conclusion that can be drawn regarding dCA when based on mean flow index methodology (Olsen, Riberholt, Plovsing et al., 2022). We have developed an open‐source publicly available R package named ‘clintools’ (Olsen et al., 2023), with the aim of simplifying the process and increasing the methodological consistency of mean flow index‐based measures.

A contributor to the findings in relation to validity and reliability of mean flow index‐based methods is the TCD technology in itself. While it has the benefit of providing non‐invasive measurements at the bedside with a very high temporal resolution, it measures linear flow velocity and not volumetric flow (Aaslid et al., 1989), and it furthermore mostly reflects regional perfusion (Svedung Wettervik et al., 2021), a limitation that is inherent for all dCA indices based on TCD. Perhaps more importantly, the same‐day and between‐day test–retest reliability as well as inter‐rater agreement of MCAv measurements obtained by TCD have all been reported to be exceedingly poor in previous studies (Loesel et al., 2009; McMahon et al., 2007; Muñoz Venturelli et al., 2017). The latter may, however, be improved by the use of automated TCD systems which are becoming more widely used, but in any event, this alone is unlikely to be the singular reason for the questionable validity and reliability of mean flow index‐based methods outlined above.

Given the grave methodological inconsistencies between previous mean flow index studies and its limitations in both construct and criterion validity as well as test–retest reliability, some inherent to mean flow index‐based methodology and others related to TCD technology, a consensus on its application in dCA studies may be needed. Transfer function analysis, another widely used dCA method, initially faced similar challenges, but since expert consensus was published in a recently updated white paper, the methodological difference between research groups has decreased markedly (Claassen et al., 2015; Panerai et al., 2023).

The mean flow index does not seem fulfil any of the requirements for a valid biomarker of biological processes (Fleming & Powers, 2012; McLeod et al., 2019). It is important that biomarkers used as surrogate markers of a biological process are both reliable and valid (Colli et al., 2014; Fleming & Powers, 2012; McLeod et al., 2019). Simple correlation analyses between clinical outcomes and biomarkers are insufficient, as a potential correlation between the biomarker and a clinical outcome may reflect disease severity and pathophysiological epiphenomena rather than being causally related to that outcome (Fleming & DeMets, 1996; Hartung, 2016). Indeed, no individual approach for data collection or for data pre‐processing stands out as superior, and because the different mean flow‐index based measures all appear both invalid and unreliable, it is questionable whether they, although theoretically appealing, can be considered markers of dCA at all.

## 8 CONCLUSION

While mean flow index‐based measures were initially considered a novelty with the potential of informing prognosis, they seems less promising almost 30 years on. Their use as measures for dCA is fraught with grave methodological inconsistencies, which renders any synthesis of the collective findings in relation to their clinical relevance meaningless. Furthermore, both the validity and the reliability of mean flow index‐based methodology is questionable and there is currently not much to suggest that any approach in terms of data collection, data pre‐processing and artefact handling will lead to measures that are physiologically or clinically relevant. Thus, caution should be exercised in any further use of mean flow index‐based measures for assessing dCA, and there is currently no sound evidence base to support their implementation in the clinical setting.

## AUTHOR CONTRIBUTIONS

Markus Harboe Olsen wrote the first draft. All authors critically revised the final draft. All authors have read and approved the final version of this manuscript and agree to be accountable for all aspects of the work in ensuring that questions related to the accuracy or integrity of any part of the work are appropriately investigated and resolved. All persons designated as authors qualify for authorship, and all those who qualify for authorship are listed.

## CONFLICT OF INTEREST

The authors declare no conflicts of interest.
